# Enhancing Capacity for Food and Nutrient Intake Assessment in Population Sciences Research

**DOI:** 10.1146/annurev-publhealth-071521-121621

**Published:** 2022-12-16

**Authors:** Marian L. Neuhouser, Ross L. Prentice, Lesley F. Tinker, Johanna W. Lampe

**Affiliations:** Cancer Prevention Program, Division of Public Health Sciences, Fred Hutchinson Cancer Center, Seattle, Washington, USA

**Keywords:** nutritional epidemiology, dietary assessment, nutritional biomarkers, regression calibration, measurement error

## Abstract

Nutrition influences health throughout the life course. Good nutrition increases the probability of good pregnancy outcomes, proper childhood development, and healthy aging, and it lowers the probability of developing common diet-related chronic diseases, including obesity, cardiovascular disease, cancer, and type 2 diabetes. Despite the importance of diet and health, studying these exposures is among the most challenging in population sciences research. US and global food supplies are complex; eating patterns have shifted such that half of meals are eaten away from home, and there are thousands of food ingredients with myriad combinations. These complexities make dietary assessment and links to health challenging both for population sciences research and for public health policy and practice. Furthermore, most studies evaluating nutrition and health usually rely on self-report instruments prone to random and systematic measurement error. Scientific advances involve developing nutritional biomarkers and then applying these biomarkers as stand-alone nutritional exposures or for calibrating self-reports using specialized statistics.

## INTRODUCTION

Adoption and maintenance of a healthy diet are among the most important and modifiable factors for ensuring healthy pregnancies (83), promoting proper growth and development in children and adolescents (38, 46), and, later in life, preventing diet-related chronic diseases such as cancer, obesity, cardiovascular disease (CVD), and type 2 diabetes (15, 58, 64, 100, 101, 103, 104). Understanding diet–health relationships is a critical public health issue, given the high morbidity and mortality from diseases related to poor diet (58, 72) and their associated health care costs (9, 34, 53, 84).

Much of the recent progress in understanding etiologic relationships between diet and health has been led by nutritional epidemiology research (48, 64, 90). Some of this research has resulted in important public policy changes, including policies that guide implementation of child and adult care food programs; food labeling laws; fortification and enrichment of fluid milk, refined grains, and salt with specific nutrients; municipal taxes on sugar-sweetened beverages as a consumption deterrent; and general population guidance for dietary intake (1, 14, 16, 23, 26, 27, 45, 55, 58). These nutrition-related policies have been intended to reduce both acute and chronic disease risk and to improve overall population health across the life span (47, 50, 58, 95, 103, 105). Likewise, nutrition advice given to individual patients by health care providers for both health promotion and disease prevention is also driven by scientific evidence generated by nutrition scientists, including nutritional epidemiologists. Clinical recommendations often follow systematic evidence reviews by the World Health Organization (35, 36), the American Heart Association and the American College of Cardiology (47, 90, 103), the American Cancer Society (88, 89), the American Diabetes Association (25), the Academy of Nutrition and Dietetics (21, 39), the World Cancer Research Fund and the American Institute for Cancer Research (104), and other professional groups and societies. In addition, these and other evidence reviews have been used by the US Preventive Services Task Force in their published nutrition-related recommendations on healthy weight and weight gain during pregnancy (22), vitamin D deficiency screening in adults (42), healthy diet and physical activity for CVD prevention in adults (41), and weight loss to prevent obesity-related morbidity and mortality in adults (19). These evidence reviews and their resulting recommendation statements are influential for individual patient recommendations, broad population guidance, and health care payors.

The strength of the evidence to improve both population and individual health is only as good as the strength of the underlying science. Herein lies an important problem. Most of the recent nutrition-related population and clinical recommendations noted above are based on self-reported diet, primarily from observational studies, and accumulating evidence supports that self-reported dietary data that do not correct for measurement error are distorted by systematic and random measurement error (67, 75, 80). The measurement error in dietary self-report leads to inconsistent evidence, which may play a role in preventing broad nutrition-focused population interventions and public policy initiatives from moving forward in a consistent manner, leaving the public confused and skeptical. Furthermore, the complexities of exposure assessment for diet may be particularly important for understanding the role of nutrition in the etiology of risk for chronic diseases, which are the leading causes of morbidity and mortality in the United States. In contrast with deficiency diseases where one nutrient is linked to one disease (e.g., vitamin C and scurvy, iron and iron-deficiency anemia), diet-related chronic diseases may result from either deficiencies or excesses of several nutrients (alone or in combination); new approaches to estimating nutritional needs for chronic disease prevention are warranted, as noted in a 2017 consensus study report from the National Academies of Sciences, Engineering, and Medicine (61).

The goal of this article is to describe the challenges of dietary assessment in population sciences research, including addressing the important problem of systematic bias and demonstrating the potential that nutritional biomarkers offer to improve the validity of dietary assessment and its application to studies of diet and chronic disease risk. We explain first the challenges of self-report dietary assessment, the strengths and limitations of standard dietary assessment methods that have been in use for decades, and the potential for nutritional biomarkers combined with novel statistical modeling to advance the field of nutritional epidemiology.

## BACKGROUND

### The Challenges of Dietary Assessment

The United States is at a critical juncture in the health of the nation, where the heavy burden of morbidity, mortality, and health care expenditures imposed by diet-related poor health must be reduced (64). The foundation for evidence-based dietary recommendations rests on obtaining accurate dietary exposure data. Assessment of diet is undoubtedly among the most complex exposure assessment problems in epidemiology. The American diet is admittedly complex and is not a single exposure. Thousands of potential food ingredients are combined either in commercial preparation or in home cooking into myriad dishes with varying additions of oils, salt, and other seasonings. Diet has recently been characterized as multidimensional (86), where one might consider exposures as nutrients (macro- and micronutrients), non-nutrient phytochemicals (e.g., flavonoids, glucosinolates, carotenoids), food groups (e.g., fruit, vegetables, grains, animal protein, dairy), individual foods (e.g., blueberries, green tea, broccoli, red meat, eggs), multi-ingredient composite foods as consumed (e.g., soups, stews, salads, tacos, pizza, hamburgers, stir fry), or other attributes related to food growing (e.g., pesticides, toxins) or preparation (e.g., heterocyclic amines, polycyclic aromatic hydrocarbons). To add to this complexity, even at a single point in time, multiple nutrients overlap within multiple food groups, with related dependencies that may be quite strong and must be considered in statistical modeling. In addition, substantial measurement error is common in self-reported nutrients and foods, with complex measurement error dependency patterns. Altogether, the challenge may seem overwhelming.

Much of the methodology in nutritional epidemiology attempts to validate specific self-report dietary assessment approaches, which seems to mean that the assessed diet is thought to be good enough for certain practical purposes, especially for disease association analyses. These validation studies have typically used some form of replicate self-report dietary assessments, or assessments using two or more different self-report assessment approaches (107). However, owing to the absence of objective intake measures in these studies, related measurement error adjustments cause investigators to inevitably make strong assumptions about at least one of the assessments, most often that one self-report assessment (e.g., multiple days of food records or 24-h dietary recalls) estimates the targeted intake aside from random measurement error that is independent of the targeted intake and independent of other pertinent participant characteristics. However, this correction strategy does not address the potential systematic measurement errors in self-reported diet that arise when individuals with certain characteristics [e.g., body mass index (BMI) >30.0 kg/m^2^ or older age] have features that differ from other persons in the study population; these characteristics influence dietary reporting, a phenomenon known as systematic bias (67, 76). Systematic bias is a serious form of measurement error because it distorts associations and is not alleviated by increasing study sample size or making simple analytic adjustments. Some form of objective measures, or at least measures that plausibly adhere to simple measurement error modeling assumptions, is needed to identify and allow for systematic bias adjustments. While considerable literature describes the use of biomarkers to examine or support the properties of specific self-report assessments (24, 56), the criteria needed to yield biomarkers that can correct complex dietary self-report measurement error for nutritional epidemiology purposes have so far received little research attention. Related developments may be able to advance the field substantially, particularly if biomarkers can be used to replace self-reported dietary intake in association studies of diet and disease outcomes.

### Current Dietary Assessment Methods: Commonly Used but Fraught with Limitations

The three most commonly used dietary assessment tools in nutrition research are the food frequency questionnaire (FFQ), food diary or record (FR), and 24-h dietary recall (24HR). The roots of these tools stem from Burke’s (12) seminal publication in 1947, “The Diet History as a Research Tool.” Born from her vision of public health nutrition research, Burke pioneered the expansion of dietary assessment from costly balance studies and controlled environment eating, which could monitor the few, to assess longer-term habitual intakes through assessing diet histories of the many with greater cost efficiency. Though her approach was more cost-efficient, Burke recognized that even with careful interviews by trained nutritionists and a review of food lists and records kept by participants, diet histories may be missing foods or could be generally incomplete. Such inaccuracies have continued to plague self-reported dietary assessment, leading to potentially erroneous research conclusions about associations between intake and health risks and to challenges in addressing patient needs in clinical care. Yet, self-reported dietary assessment remains the stanchion of public health nutrition research and clinical care.

### Food Frequency Questionnaires

FFQs are food lists with options for respondents to mark foods and food combinations, frequency of consumption, and approximate portion size typically consumed over a period of time, which may range from “in the past month” to “in the past year.” Being self-administered on a mark sense print format or online, FFQs are a cost-effective and low-burden method for capturing a respondent’s usual or average dietary intake over the specified time period. FFQs are popular for reporting dietary intake in large population sciences research studies.

Designing FFQs requires careful attention to the characteristics of the population under study (43), including age, literacy, language, cognitive abilities, and representative foods and preparation methods in order to capture both intraindividual and interindividual intake variation. Block et al. (8) in 1986 describe in detail a data-based approach for designing what was then referred to as food histories and are often now known as FFQs. In 1986, Block et al. published the nutrient sources from foods consumed commonly in the United States (8). These publications aided the design of FFQs, including the FFQ used in the Women’s Health Initiative (WHI) (106).

The WHI, conducted nationally in 40 clinical centers across the United States (106), offers a useful case study for designing an FFQ (69). After selecting commonly consumed foods, frequency, and portion options for inclusion in the WHI FFQ, the design team attended to cultural and regional foods. A feature of the WHI FFQ was that it captured dietary fat intake change to be responsive to the WHI Dietary Modification Trial, which compared a usual diet group to a low-fat dietary pattern intervention group (87). To accomplish this task without becoming overly lengthy, the WHI FFQ began with a series of adjustment questions that asked about types of dairy, e.g., nonfat, low-fat, and regular dairy, cooking methods, and at-the-table additions of various types of fat, including no fat, oils, butter, etc. The adjustment questions were linked to the line-item analysis to add precision to the line-item estimates (69). In studies where FFQs are designed for specific populations or research questions, the FFQs might not be transferrable among studies or research questions. Although less burdensome to respondents than completing multiple-day FRs or 24HRs, FFQs are not necessarily considered by all to be easy to complete (13).

### Food Diary or Food Record

The terms food diary and food record are functionally synonymous, so here we use the term food record (FR). FRs are designed to capture short-term intake of foods and portion sizes actually consumed. The number of days reported for FRs depends on whether individual or group intakes are being assessed, the nutrients of interest, and the frequency and types of foods (5), although three to seven days are most commonly completed. FRs typically cover one week’s intake with days fewer than seven alternating to capture varied days of the week (e.g., weekdays, weekends) and variable times of day to cover variable schedules and to lessen the correlation of consecutive day intake. The burden of completing an FR is greater than that of completing an FFQ. To complete an FR, the respondent keeps track of and records their food and beverage intake for a specified number of days. Respondents receive training, measuring utensils, and often an instructional booklet with portion photos, and review by study staff is common.

### 24-Hour Recalls

24HRs capture an individual’s intake over a 24-h period. Similar to FRs, 24HRs capture short-term dietary intake of foods and portion sizes actually consumed. The number of days recalled depends on whether variation within individual intake is in question, in which case multiple days are needed to calculate intraclass correlations, or group means are in question, in which case a single 24HR from the group or select subset group is needed (4). Furthermore, the number of days may depend on particular foods or nutrient exposures of interest. For example, when foods or nutrients of interest may be less commonly consumed, such as fish or omega-3 fatty acids, more recalled days may be needed. As with FRs, three or four days covering varying days of the week are common.

Standard 24HRs are administered by trained interviewers, often registered dietitians using the US Department of Agriculture (USDA)’s five-step multiple-pass system (85) to define, refine, and quantify intakes. The five steps include a quick list of foods consumed the previous day, a review to collect possibly forgotten foods, time and location cues, details about intake, and a final review (17, 18). 24HRs may be conducted by automated systems on mobile devices (93) or online (98).

### Paperless Forms of Dietary Self-Report: Automated, Mobile, Online

Dietary self-report, like many other research data collection activities, are going or have gone paperless. Examples include online Automated Self-Administered 24-Hour Dietary Recall (ASA-24) (98) and several mobile device applications as summarized by Schembre and colleagues (93). The premise behind digital collection of dietary data is the same as traditional dietary assessment but may be streamlined for both study participants and staff. There is little evidence that digital collection of dietary data reduces or eliminates the well-known systematic error that can profoundly affect dietary self-report (40).

### Nutrient Analysis

All dietary assessment instruments must be analyzed with high-quality food and nutrient databases. The processing of the raw food consumption data is linked with food composition databases to yield an output of the average daily intake of more than 150 nutrients (macronutrients, micronutrients, energy), food components (e.g., added sugars), plant compounds (e.g., carotenoids, isoflavones), and food group serving counts (fruits, vegetables, whole grains, refined grains, animal protein, plant protein). Comprehensive research-grade food and nutrient databases must be used, as most foods have been subjected to chemical analysis to derive nutrient composition. In the United States, these include (*a*) the USDA National Nutrient Database for Standard Reference, Legacy Release (https://data.nal.usda.gov/dataset/usda-national-nutrient-database-standard-reference-legacy-release), which has data on 150 output variables from nearly 7,800 foods commonly consumed in the United States; and (*b*) the NDSR^®^ Food and Nutrient Database (http://www.ncc.umn.edu/ndsr-database-page/), which includes 178 output variables from 19,500 foods. Food composition databases should include fresh raw ingredients, ready-to-eat foods, and restaurant foods.

### Limitations of Dietary Self-Reports

Despite their common use, self-report dietary assessment tools are fraught with measurement error and our work has shown that the measurement error is common across all three tools (FFQ, FR, and 24HR) (76). Random measurement error or bias may be alleviated somewhat by a larger sample size; however, the more troublesome source of error is systematic bias, which may be influenced by respondents not being familiar with portion sizes, not knowing food ingredients when not in control of their own cooking, social desirability, or personal characteristics (67). In 2003, Subar et al. (97) reported, from the Observing Protein and Energy Nutrition (OPEN) study of nearly 500 men and women, that men on average underreported energy intake by up to 14% on 24HR and 36% on FFQs; greater underreporting was noted with higher BMIs, compared with a well-established biomarker for total energy intake, i.e., doubly labeled water assessment. Protein was also found to be underreported, though to lesser degrees, when compared with the well-established 24-h urinary nitrogen (UN) biomarker (7). A few years later, Neuhouser et al. (67) reported similar results from a subsample of 540 participants from the WHI, adding findings of systematic underreporting of energy and protein as body mass increased as well as systematic bias by age and race/ethnicity (67). Prentice et al. (76) reported similar findings among a sample of 450 participants from the WHI observational study for FFQs, FRs, and 24HRs. Pooling data from five cohorts (including those from OPEN and WHI) in the United States representing 2,265 men and women showed similar results (28). Systematic bias has also been reported for nutrients, including sodium and potassium, which can be reliably measured with recovery biomarkers in 24-h urine collection samples (29, 74).

## DIETARY BIOMARKERS

Understanding how foods ingested are linked to human physiology, metabolism, and health outcomes must move beyond error-prone self-report. Biomarkers of diet and their effects on host physiology and disease risk are an integral part of nutrition research, and their use in research may provide a more rigorous scientific approach compared with methods currently used. Diet-related biomarkers are often classified into three groups: (*a*) exposure biomarkers, (*b*) susceptibility markers, and (*c*) outcome biomarkers (56). Exposure biomarkers are those that directly reflect food intake. Examples of compounds that come from diet exclusively, since they are not endogenously made (or made at very low levels), include UN (protein intake), plasma carotenoids (fruit and vegetable intake), and plasma long-chain omega-3 fatty acids (fish intake). Susceptibility biomarkers are those that are not direct makers of the intake of specific foods but are made endogenously in response to the metabolism of foods ingested, and they function as disease susceptibility biomarkers. Exposure biomarkers, providing objective measures of dietary intake of particular nutrients or food groups (92), have been a primary focus of research to improve dietary assessment in nutritional epidemiology. Examples of susceptibility biomarkers include serum cholesterol in response to saturated fat intake (90) and serum triglycerides in response to high carbohydrate intake (3, 68). Outcome biomarkers are those that might be monitored in a dietary intervention trial for adherence, those that are the primary outcome to assess dietary change, or those that offer etiologic insight regarding diet and metabolism in short-term trials, such as controlled feeding trials (62, 66, 91).

### Established Biomarkers

Dietary biomarker development has generally followed the trajectory of the field of nutrition science. The early years of nutrition science were focused on the essentiality of nutrients to prevent classic nutrient deficiency diseases; biomarkers have been used for both population surveillance and individual status assessment with respect to essentiality, including hemoglobin or hematocrit to assess iron-deficiency anemia and serum retinol to assess vitamin A deficiency, which causes night blindness and immune dysfunction (51, 70, 99). These biomarkers have been useful tools to monitor status and prevent deficiencies. More recently, the field of nutrition science has recognized that the majority of nutrition-related diseases today may result from both over- and underconsumption, and thus new tools are needed to quantitatively assess intake more effectively (61). Unfortunately, there are few established quantitative dietary intake biomarkers that can be used in this manner. Quantitative biomarkers include recovery biomarkers that reflect absolute intake over a defined period of time: Doubly labeled water (DLW) uses ingestion and excretion of two stable isotopes, deuterium and O_18_, to quantitatively measure total energy expenditure (94), and hence total energy intake among weight-stable persons, and UN from 24-h urine collections as a measure of total protein intake (7). In addition, 24-h urine recovery of sodium and potassium has been used as a biomarker of sodium and potassium intake (37), although considerable random error may attenuate these measures, especially for sodium, and certain systematic biases have also been postulated (37, 59, 74). Concentration biomarkers (i.e., blood concentrations of vitamins, phytochemicals, lipids, or their metabolites) are correlated with intake but cannot be readily used to calculate an absolute level of intake. These biomarkers make up the majority of potential analytes available for characterizing dietary exposure (44, 57).

The established recovery biomarkers have been compared with self-reported diet, as assessed using FFQs, FRs, or 24HRs in various nutritional biomarker studies, and with meta-analyses (2, 28, 30, 37, 67, 76, 107). In addition to the substantial random measurement error that is evident from replicate applications of the self-report tools to individual study participants, these studies and meta-analyses also reveal substantial systematic biases. Briefly, self-reported energy is very poorly estimated by any of the major dietary assessment methods, but the correspondence with DLW energy can be much improved through novel statistical methods by developing calibration equations that include such participant characteristics as age, self-reported race/ethnicity, and BMI. For example, overweight and obese individuals at the upper end of the BMI distribution tend to underreport total energy intake by about 30–40%, whereas there is little underreporting among normal-weight persons in the populations studied (67). Total protein intake is also underestimated by self-report, whereas protein density (i.e., total protein/total energy) is overestimated (67), and in general may be less affected by other sources of systematic bias. Sodium is poorly estimated using any of the self-report methods; sodium density estimation appears to have systematic bias related to BMI, while potassium is considerably better estimated by self-report (74). The relationship between sodium and sodium density self-reports that correspond to putative biomarker assessments can be somewhat improved by bringing participant characteristics into calibration equations, but the calibrated intake estimates still do not correlate strongly with actual intake, as shown in a controlled feeding study (44).

Nutrient biomarkers, while extensively studied, are limited in their capacity to capture the breadth and complexity of dietary intake at the level of foods, food groups, and dietary patterns. With the shift in nutrition research from the study of essential nutrients to the role of nonessential food constituents in the prevention of chronic disease, dietary biomarkers have also shifted to include compounds associated with particular foods or classes of foods. The classification of exposure biomarkers (which could be single compounds or a panel of compounds) has also expanded to include food-component biomarkers (nutritive and non-nutritive), biomarkers of food intake, and dietary pattern biomarkers, while recognizing that some markers may be used for characterizing a variety of diet exposures (31). For example, plasma carotenoid concentrations are effective biomarkers of carotenoid intake (44), fruit and vegetable intake (44), and adherence to a healthy dietary pattern (49, 54).

### Unresolved Issues

These studies of recovery-based nutritional biomarkers raise important questions: Is the paucity of established biomarkers that can be used quantitatively as recovery biomarkers a necessity, or can additional biomarkers be identified by also considering new measures in blood or other body fluids and by bringing in higher-dimensional data sources, such as serum and urine metabolomics profiles? Trying to answer these questions about nutritional biomarker discovery and their application to population sciences research has been the aim of considerable recent research (56). Furthermore, which properties should be required for a suitable dietary intake biomarker in the nutritional epidemiology area? This important topic has received very little discussion in the literature to date but very much needs rigor and reproducibility, including for newly identified metabolomics-based biomarkers (72).

The extent to which the properties of intake biomarkers depend on participant characteristics are transportable to other populations is also an important concern. At this stage of biomarker development, it seems advisable for cohort-based research groups to incorporate biomarker development studies for their populations, at least if the research includes a substantial focus on nutritional exposures and their associations with chronic disease risk. The reasoning is that the participant characteristics associated with systematic bias in dietary self-report will vary in each study population. In addition, it will typically not be practical to measure established or novel dietary biomarkers for all members of large epidemiologic cohorts owing to the extensive costs and logistics involved. Rather, such biomarkers may be measured in nutritional biomarker substudies in large cohorts and used to develop calibration equations for use in calculating biomarker-calibrated intakes for participants in the entire cohort for use in disease association analyses. This raises the question of which properties should be required for calibrated intakes, a topic of ongoing research (44, 72).

## PROMISING ADVANCES IN BIOMARKER DEVELOPMENT

Metabolomics, the high-throughput study of substrates and products of metabolism in a biological system, is a promising application for nutritional biomarker development (10, 92). It involves the measurement of hundreds to thousands of small molecules in biofluids or tissues by mass spectrometry (MS) and/or nuclear magnetic resonance spectroscopy (NMR), coupled with various dimensionality-reduction methods for multivariate analysis and application of classification, regression, and/or prediction methods for analysis (60, 63, 81). Overall, metabolomic analysis of biospecimens can provide a comprehensive reflection of exposures, such as diet, as well as of the host and gut microbial metabolic responses to these exposures and the impact of host and gut microbial metabolic phenotype on biomarker metabolism (10). This complexity is both a challenge and an opportunity. On the one hand, it means that, in most cases, metabolomic biomarkers of intake of a food or food pattern are unlikely to be coming solely from the food itself. On the other hand, it means that metabolomics data are providing a richer snapshot, including the biochemical and physiologic effects of dietary exposures on biological pathways related to health and disease outcomes. The development of robust metabolomics platforms has led to several initiatives to discover and validate new metabolomics biomarkers of diet (10, 56). While many metabolomics biomarker studies have relied on cross-sectional study designs and self-reported dietary intake, with its inherent limitations, more robust dietary intervention approaches have also been used for dietary biomarker discovery and validation. Examples are given below.

### The Nutrition and Physical Activity Assessment Study

In these WHI ancillary studies, efforts to identify and apply objective dietary biomarkers to strengthen the evidence of diet–disease associations using biomarker-calibrated intake have expanded from using established nutrient recovery biomarkers (6, 67, 74, 78) to using metabolomics-based biomarkers (109, 110). One component of the Nutrition and Physical Activity Assessment Study (NPAAS) was a controlled feeding study (NPAAS-FS) designed specifically for dietary biomarker development, which was conducted in a subset of 153 WHI participants who were fed their habitual diets as part of a controlled feeding study protocol (44). In this unique feeding study design, the approach aimed to preserve the normal variation in nutrient and food consumption for each participant and minimize short-term perturbation of the biomarkers of intake. Blood and urine collected at the end of a two-week period were analyzed using targeted liquid chromatography (LC)-MS/MS (serum), lipidizer/differential mobility spectrometry (serum), gas chromatography (GC)-MS (urine), and NMR (urine) metabolomics platforms. To date, these data have been applied to develop metabolite signatures for macronutrient intakes (e.g., protein and animal protein, carbohydrate, and dietary fiber) (77, 79), as well as intakes of groups of foods (e.g., meat) (110), and plans are underway for dietary patterns.

Several strategies have been used for biomarker variable selection using the NPAAS-FS multiplatform metabolomics data. For an agnostic approach, linear regression models, with least absolute shrinkage and selection operator (LASSO) methods for variable selection (96), were run for the regression of each (log-transformed) macronutrient intake variable (derived from the consumed menus) on all the metabolites; fivefold cross-validation was used to select a penalty parameter to limit the number of variables to fewer than 15. The prediction model was built with a second round of linear regression after variable selection (109). For a more targeted approach, literature reports of metabolite correlates of meat consumption were used to select metabolites of meat-intake biomarkers for testing in linear regression models (110). The established recovery biomarkers, log-transformed DLW energy and UN-based protein, provided a benchmark for acceptable biomarkers, and a regression equation with cross-validated R^2^ (CV-R^2^) of ≥36% was selected as a criterion for acceptable intake biomarkers in the NPAAS-FS. The rationale is that new biomarkers should have a rigorous correlational criterion that approximates the criterion observed for gold standard DLW and UN biomarkers. Investigators have developed a pipeline to apply the biomarkers to calibration of self-reported intake within the WHI cohort. As part of this pipeline, log-transformed metabolite signatures meeting the cross-validation R^2^ were advanced to the next step of regressing linearly on corresponding log-transformed FFQ intake values and personal characteristics to produce calibration equations for these variables. These equations were then used to calculate calibrated intake values in the WHI cohorts and associate these with disease risk in the WHI cohorts (77, 79, 110).

### The Food Biomarker Alliance Program

Larger initiatives have also contributed substantially to dietary biomarker discovery using metabolomics. From 2014 to 2018, the Joint Programming Initiative A Healthy Diet for a Healthy Life Food Biomarker Alliance (FoodBAll) a consortium of 24 partners from 13 countries, carried out a systematic exploration and assessment of metabolomics-based biomarkers of food intake (11). With the goal of providing a better assessment of the food intake in different European regions, well-defined, standardized, short-term intervention studies were conducted in several centers to identify potential food intake biomarkers of a variety of foods, including sugar-sweetened beverages, apple, tomato, banana, milk, cheese, bread, meat/meat products, red meat and white meat, potato, carrot, peas, lentils, beans, and chickpeas. These studies have identified potential urinary and blood biomarkers of exposure to specific foods, generated information about the time-response effect of these putative biomarkers, and contributed to the development of biomarker calibration equations for some foods (32). For example, the dose–response relationship of 24-h urine proline betaine with orange juice intake was established in a controlled feeding study (33) and subsequently used to derive the optimal biomarker calibration equation in a cross-sectional study, testing different functional specifications and biomarker transformations (20). While this application used an established, reliable single-metabolite biomarker, ideally this approach could also be applied to more complex scenarios where foods require multi-metabolite signatures.

As part of the FoodBAll program, researchers established guidelines for conducting literature searches on food biomarkers so as to identify existing candidate biomarkers for a specific food or food group and to provide available evidence for the subsequent systematic evaluation of the quality of such compounds as biomarkers (71). A recent review of 244 studies identified 69 metabolites that were classed as potentially useful biomarkers of food intake, covering fruits, vegetables, meat, seafood, legumes, coffee, and high-fiber foods (82). This procedure is expected to help prioritize further future work on validating putative biomarkers. Nonetheless, it also reinforced that specific biomarkers will not necessarily be found for every type of food and that complex modeling approaches may be needed for appropriate selection of combinations of multiple metabolite biomarkers (i.e., metabolite signatures), while accounting for possible confounders (102). FoodBAll investigators also established criteria for validating identified biomarkers; these criteria include assessment of biologic plausibility, time and dose response, robustness, reliability, stability, and analytical performance of the method used to measure them (24).

### The Dietary Biomarker Development Consortium

In 2021, the National Institutes of Health (NIH) and the USDA’s National Institute of Food and Agriculture established the Dietary Biomarker Development Consortium (DBDC; https://dietarybiomarkerconsortium.org/), comprising three clinical centers and a data coordinating center, to expand the available metabolite signatures for foods within the USDA MyPlate food groups (https://www.choosemyplate.gov/). MyPlate operationalizes the US Dietary Guidelines for Americans to help consumers choose fruits, vegetables, grains, protein foods, and dairy as part of their overall dietary pattern. Multidisciplinary teams within the DBDC provide the necessary expertise and experience in dietary intervention and dietary assessment, metabolomics, and bioinformatics for exploring metabolomics-based dietary biomarkers and biostatistical analysis of dietary biomarker data. The clinical centers are generating initial sets of metabolite signatures for selected foods in the context of controlled feeding in small groups of study participants. Properties of the identified metabolites, such as pharmacokinetic profiles and half-lives, as well as initial performance of the signatures will be evaluated in this phase. In follow-up studies, biomarker signatures will be evaluated further for predicting recent and habitual consumption of more complex mixed meal diets and dietary patterns fed as part of controlled feeding studies. The performance of the biomarkers will also be compared with the current benchmark predictive markers (e.g., 24-h UN, DLW, urinary sodium). Robust validated markers will ultimately be tested for their ability to predict both recent and habitual consumption of dietary components in independent cohorts along with 24HRs and FRs and other benchmark biomarkers.

Progress to date suggests that metabolomics data may not only generate novel biomarker signatures of diet but also help to expand and improve on methodologic approaches, yielding novel, robust protocols for dietary biomarker discovery and validation. Much of the focus has been on the discovery of metabolomic biomarkers for specific nutrients or foods; biomarkers of dietary patterns and the next steps for biomarker validation and calibration of self-reported dietary assessment tools in diverse populations remain less well studied. For full realization of the application of metabolomics to dietary biomarker development, rigorous biomarker validation, as well as the methodological and technological challenges inherent in the metabolomics field, still needs to be addressed (10, 56).

## SPECIALIZED STATISTICAL MODELING FOR BIOMARKERS AND APPLICATIONS TO CHRONIC DISEASE RISK

**Figure 1:**
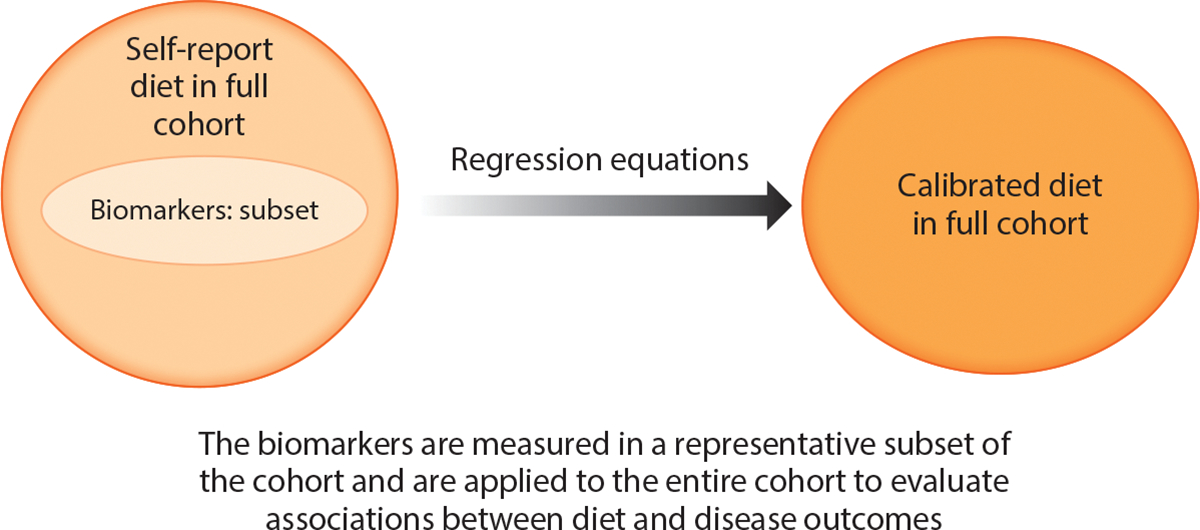
Nutrient biomarker calibration approach where biomarkers are collected in a representative subset of the entire cohort and applied to the entire cohort using regression calibration.

Despite the promise for nutritional biomarkers to improve dietary assessment in population studies, it is neither practical nor financially feasible to assess biomarkers in cohorts with tens of thousands of participants. Related to these financial and logistical limitations is the complication that the DLW energy intake biomarker requires an expensive protocol and cannot be measured from biospecimens in biorepositories owing to the need to ingest the stable isotopes and collect timed urine samples over a course of several days to two weeks. For these reasons, cohort substudies may be the most practical way to implement nutritional biomarker assessments. In our work in the NPAAS, the biomarkers together with the participant characteristics are used to develop a calibration equation where participants without biomarker values are given a predicted energy intake value representing true energy intake, given a participant’s age, BMI, race/ethnicity, and other characteristics (67). In this way, the objective biomarkers are efficiently used to create predicted intake values for the entire cohort (Figure 1). Thus, the biomarker values can be used as response variables in a second set of regression equations that aim to reexpress the biomarkers in terms of self-report dietary data and individual participant characteristics, measures that are available for the members of the larger epidemiologic cohorts. The resulting calibration equations will be developed by regressing biomarkers for the set of log-transformed biomarker intakes on corresponding log-transformed dietary self-report assessments and participant characteristics, and a second R^2^ criterion will be needed to aid in the evaluation of the calibration equation. For example, a 36% adjusted R^2^ has been applied as a criterion in WHI calibrations to date, with adjustment that reduces the influence of biomarker noise on the regression R^2^ value (65, 77, 109). Fortunately, the biomarker and calibration correlational (R^2^) criteria can be applied essentially independently relative to statistical properties of the resulting biomarker-calibrated intakes. Under pertinent modeling assumptions, the set of calibrated dietary intakes, along with potential confounding factors, can be inserted into standard outcome analyses, such as Cox regression, for time to clinical outcomes in cohort settings, with some refinement needed for variance estimators to acknowledge random variation in calibration equation coefficient estimates (72, 73). Finally, while the biomarker and calibration R^2^ criteria ensure a certain closeness between estimated and actual intakes, it is also important to consider possible influential sources of reduced sensitivity or specificity for both biomarkers and calibrated intakes, even if evaluation of sensitivity and specificity can be done only informally in specific applications.

These analytic approaches using statistical calibration have revealed an important role for total energy intake in CVD, cancer, and diabetes incidence and mortality analyses (108) because, for the first time, energy can be used in a mostly unbiased manner as opposed to self-report. However, these analyses are limited by a strong dependence of DLW-calibrated total energy intake on BMI. In fact, these disease associations tend to decrease or may even disappear following separate control for BMI in outcome modeling. The question of whether BMI, or other measures of body fat accumulation, should be regarded as a mediator, confounder, or both in health-related analyses remains outstanding and represents one of the most important issues to be resolved in nutritional epidemiology going forward. Resolution of these issues is especially important because the vast majority of nutritional epidemiology reports express dietary intakes as ratios to total energy intake, while total energy intake, which is poorly assessed using any of the self-report dietary assessment measures, remains largely unstudied. To address this limitation, one may consider a cohort study design with biomarkers, including for total energy, and self-report data available longitudinally over major stretches of the life course. Alternatively, concerted efforts to develop a total energy biomarker that can be derived from stored specimens could be considered, as such a biomarker could open up more efficient study designs to address this important total energy and health topic using a biomarker instead of self-report.

## SUMMARY POINTS

1. Dietary intake over the life course is strongly associated with one’s probability of healthy aging on the one hand and risk of costly, diet-related chronic diseases, such as obesity, cancer, type 2 diabetes, and CVD, on the other hand. Some of these diseases, obesity and type 2 diabetes in particular, emerge in childhood and adolescence, bringing much needed urgency to finding population-based solutions. Because dietary recommendations provided to both clinicians and the public are evidence based, the research community must provide stronger, more rigorous nutrition research to fully understand and model very complex and multidimensional food intake.
2. Much of the past research, including that used for national policies and guidelines, has relied on self-report of diet, which is subject to substantial random and systematic measurement error. Error-prone results are neither rigorous nor reproducible—one of the standards used to evaluate science.
3. Biomarkers of diet, when evaluated with high standards for rigor and reproducibility, may be used to calibrate or adjust the measurement error–prone self-report. In some cases, the biomarkers may be used as direct exposures, avoiding reliance on self-reported diet.

## FUTURE ISSUES

1. We recommend that (*a*) a broad array of foods and their combinations in dietary patterns are represented in biomarker research (56), (*b*) standardized approaches are established to support biomarker discovery and validation, and (*c*) statistical and computational approaches are developed to best model diet complexity (52, 56). The FoodBAll program in Europe and the Dietary Biomarkers Development Consortium in the United States, which are both discovering and validating metabolomics biomarkers of foods, are a step in the right direction, as are the recently initiated efforts of the NIH’s Precision Nutrition for Health.
2. The research community and federal funders should further strive to provide the resources necessary to support this important endeavor. The health of the nation and prevention of diet-related diseases deserve the most definitive science possible. Much of the needed research will necessarily involve observational designs, but there also needs to be a substantial provision for randomized, controlled dietary intervention trials when the related hypotheses and interventions are sufficiently well developed and public health implications are sufficiently strong.

## Abbreviations

CVD: cardiovascular disease
FFQ: food frequency questionnaire
FR: food record
24HR: twenty-four hour recall
WHI: Women’s Health Initiative
UN: urinary nitrogen
DLW: doubly labeled water
NPAAS: Nutrition and Physical Activity Assessment Study
